# Effects of aquatic exercise program versus on-land exercise program on cancer-related fatigue, neuropathy, activity and participation, quality of life, and return to work for cancer patients: study protocol for a randomized controlled trial

**DOI:** 10.1186/s12906-024-04367-8

**Published:** 2024-02-02

**Authors:** Michal Nissim, Yakir Rottenberg, Naama Karniel, Navah Z. Ratzon

**Affiliations:** 1 Teachers for Students With Complex and Multiple Disabilities Track, The David Yellin Academic College of Education, Jerusalem, Israel; 2 Department of Oncology, Hadassah Medical Organization and Faculty of Medicine, Hebrew University of Jerusalem, Jerusalem, Israel; 3 Physiotherapy Department at Hadassah Medical Organization, Jerusalem, Israel; 4 Sackler Faculty of Medicine, Department of Occupational Therapy, School of Health Professions, Tel Aviv University, Tel Aviv, Israel

**Keywords:** Aquatic therapy, Cancer, Participation and activity, Quality of life, Return to work

## Abstract

**Background:**

Exercise has shown positive effects on fatigue, exhaustion, neuropathy, and quality of life in cancer patients. While on-land exercises have been studied, the aquatic environment offers unique advantages. Water's density and viscosity provide resistance, enhancing muscle strength, while hydrostatic pressure improves venous return. This trial aims to investigate the effect of aquatic exercises on time to return to work, work hours, work-related difficulties, daily life activity and participation, quality of life, exhaustion, fatigue, and neuropathy among cancer patients, compared to on-land exercise intervention group and a non-exercise group.

**Methods:**

This randomized controlled trial will include 150 cancer patients aged 18–65 years with stage III colon cancer or breast cancer patients with lymph node involvement. Participants in the aquatic exercise intervention group will undergo an 8-week, twice-weekly group-based Ai-Chi program, while the on-land exercise group will perform identical exercise. The control group will not engage in any exercise.

The primary outcome will be assessed using an employment barriers questionnaire, capturing return to work date and working hours and daily life participation and activity and quality of life. Secondary outcomes include exhaustion, fatigue, and neuropathy. Data will be collected at baseline, post-intervention (8 weeks), and at 3,12, and 24 months. Mixed variance analyses will explore relationships among groups and over time for independent variables, with separate analyses for each dependent variable.

**Discussion:**

The potential benefits include an earlier return to work for patients, reducing their need for social and economic support. The study's implications on socio-economic policies are noteworthy, as a successful intervention could offer a cost-effective and non-invasive solution, improving patients' quality of life and increasing their participation in daily activities. This, in turn, could lead to a faster return to work, contributing to both personal well-being and broader societal interests by reducing reliance on social services.

**Trial registration:**

The trial is registered at ClinicalTrials.gov NCT05427344 (22 June 2022).

**Supplementary Information:**

The online version contains supplementary material available at 10.1186/s12906-024-04367-8.

## Introduction

### Background and rationale {6a}

The number of patients being treated for cancer continues to grow [1], which presents a challenge to treatment centers, health and welfare systems, and to the patients themselves. The majority of cancer patients experience exhaustion and fatigue [2, 3], as well as neuropathy [4, 5], as a consequence of their treatments. These factors adversely impact their participation in daily activities and reduce their quality of life [6, 7]. Additionally, they may affect the ability to function at work and delay a return to employment [8, 9].

Clinical studies have demonstrated that physical activity has a positive effect on the levels of exhaustion and fatigue [10], as well as neuropathy [11], and the general quality of life [12] of cancer patients. Consequently, patients being treated for cancer are advised to avoid inactivity [10, 13]. Paradoxically, this may lead to a scenario where exhaustion and fatigue contribute to decreased physical activity, while decreased physical activity increases exhaustion and fatigue [14], and may even be associated with a decreased quality of life among cancer patients [15, 16]. Therefore, it is important to determine the type of exercise that is most beneficial for patients and encourages them to persevere.

Most previous studies have focused on the effect of exercise on land [17]. For example, various literature reviews [18] have described the practice of Tai Chi as beneficial in reducing exhaustion and fatigue to some extent, and have suggested that it may improve the quality of life of cancer patients. The environment in which physical activity takes place is important. In this context, the properties of water and their effect on the submerged human body may be particularly beneficial for cancer patients. The density and viscosity of the water provide muscle resistance and thus improve muscle strength, while the hydrostatic pressure improves venous return, and immersion in water permits motility that is not possible on land [19]. Previous studies among breast cancer patients reported that exercise in water reduced fatigue [20, 21]. However, there is not much evidence for this topic. It is possible that the combination of the advantages of an aqueous environment and those of Tai Chi may be beneficial to cancer patients, and this represents the focus of the present study. The practice of Tai Chi in water was developed by Jun Konno and is termed Ai Chi [22]. Participants in Ai Chi perform 19 movements based on the Tai Chi Chuan technique while immersed in water to shoulder height [23].

### Objectives {7}

Based on the literature review described above, the objectives of the present study are:

(A)  To examine the effects of an 8-week physical activity program in water, on time to return to work, work hours, perception of work-related difficulties, and work absenteeism, among cancer patients, as compared to an intervention group undergoing identical physical exercise on land, and a third group with no additional exercise.
(B)  To examine the effects of an 8-week physical activity program in water on activity and participation in the daily life and quality of life of cancer patients compared with the other two intervention groups (the same physical activity on land and the group without further physical exercise).
(C)  To examine the effects of an eight-week water exercise program on exhaustion, fatigue, and neuropathy in cancer patients compared with the other two intervention groups (the same physical exercise on land and the group without further physical exercise).

### Trial design {8}

This is the protocol of a parallel-group, controlled, randomized trial, with a pre-post and repeated follow-up measures between-group design.

**Fig. 1:**
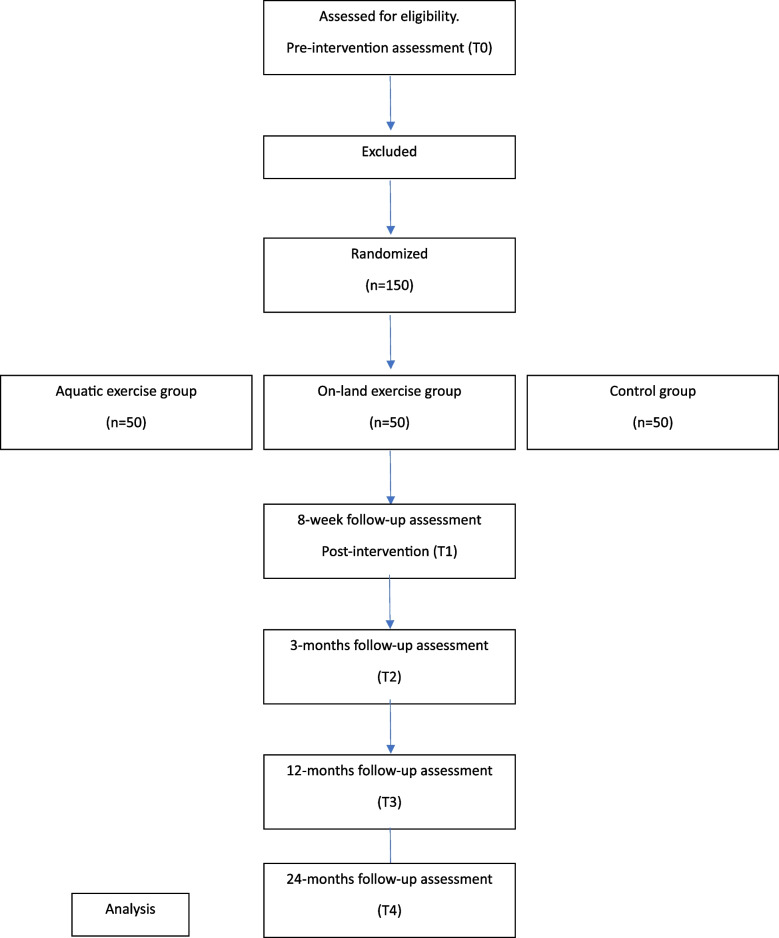
CONSORT flow diagram

A three-arm trial is going to be carried out. Participants who meet the recruitment criteria bellow will be randomly assigned to Group 1: Water exercise group, Group 2: Land exercise group, or Group 3: Control group. The assessment will be conducted at five-time points: an initial assessment before being randomized (pretreatment; T0), at the end of the intervention (post-treatment; T1), 3 months after the completion of the intervention (T2), 12 months after the completion of the intervention (T3), and 24 months after the completion of the intervention (T4). CONSORT patient’s flowchart is presented in Fig. 1.

## Methods: Participants, interventions and outcomes

### Study setting {9}

The trial will take place at Tel Aviv University and the Hadassah Medical Center hydrotherapy pool, Israel.

### Eligibility criteria {10}

Criteria for inclusion in the study will be: (a) age between 18–65; (b) diagnosis of cancer; and (c) a score above 26 on the cognitive MOCA test [24]. Volunteers with orthopedic injuries that in the opinion of the attending physician prevent their participation in physical exercise, or individuals engaged in other physical activity on a regular basis, will not be eligible to participate in the present study. Recruitment of colorectal cancer patients and breast cancer patients for the study will be within 3 months of cessation of chemotherapy treatment.

### Who will take informed consent? {26a}

At the initial screening, participants will receive informed consent from their doctor and will have the opportunity to ask questions about their participation. After that, they will receive the informed consent form and will ask to sign it.

### Additional consent provisions for collection and use of participant data and biological specimens {26b}

In this study, biological samples will not be collected. Participant data will be used only for the research proposes.

### Interventions

#### Explanation for the choice of comparators {6b}

The land exercise group is chosen in order to control for the environmental aspects of the intervention and the control group was chosen in order to control for the motor aspect of the intervention.

### Intervention description {11a}

- (A) Water exercise group where the Ai-Chi technique was selected as the exercise method of choice. The activity will take place in a hydrotherapy pool approved by the Ministry of Health and will be led by a hydrotherapist who has been trained in Ai-Chi.
- (B) Land exercise group who will perform the same Ai-Chi movements on land in order to standardize the groups. The activity will take place in a hall and will be led by a physiotherapist who has been trained to teach Ai-Chi on land.
- (C) Control group who will not perform additional physical activity or receive any extra treatments. In accordance with the recommendations for physical activity among cancer patients and the guidance of the hydrotherapy organization for hot water activities, the exercise program will be scheduled for 30 min twice a week for 8 weeks. The physical activities will take place in groups of 5 participants per group.

### Criteria for discontinuing or modifying allocated interventions {11b}

In this study, the criteria for discontinuing the intervention will be (1) participants who fail to attend two or more sessions, and (2) participants who request to withdraw from the trial.

### Strategies to improve adherence to interventions {11c}

The therapists of intervention groups will encourage the participants to attend intervention sessions. If a participant is absent from a session, a research member will contact personally.

### Relevant concomitant care permitted or prohibited during the trial {11d}

In this trial, participants who receive another intervention during their participation in the study will be excluded.

### Provisions for post-trial care {30}

No potential harm is expected from this study.

### Outcomes {12}

Data will be collected by a medical student in the fourth year of studies at the following time points: before the start of the intervention (at baseline), after 8 weeks of intervention, and then at 3, 12, and 24 months from the end of the intervention.

### Primary outcome

1. Employment Barriers Questionnaire [25] and Date of return to work and duration of working hours—self-report by the study participants.
2. WHODAS 2.0—to examine the degree of activity and participation in daily life [26].
3. EORTC QLQ-C30—to evaluate the quality of life from the point of view of the participant [27].

### Secondary outcomes

1. Piper Fatigue Scale—to assess the levels of exhaustion and fatigue [28].
2. Neuropathy Questionnaire (EORTCOLO QLQ-CIPN20) [27].

### Participant timeline {13}

**Fig. 2:**
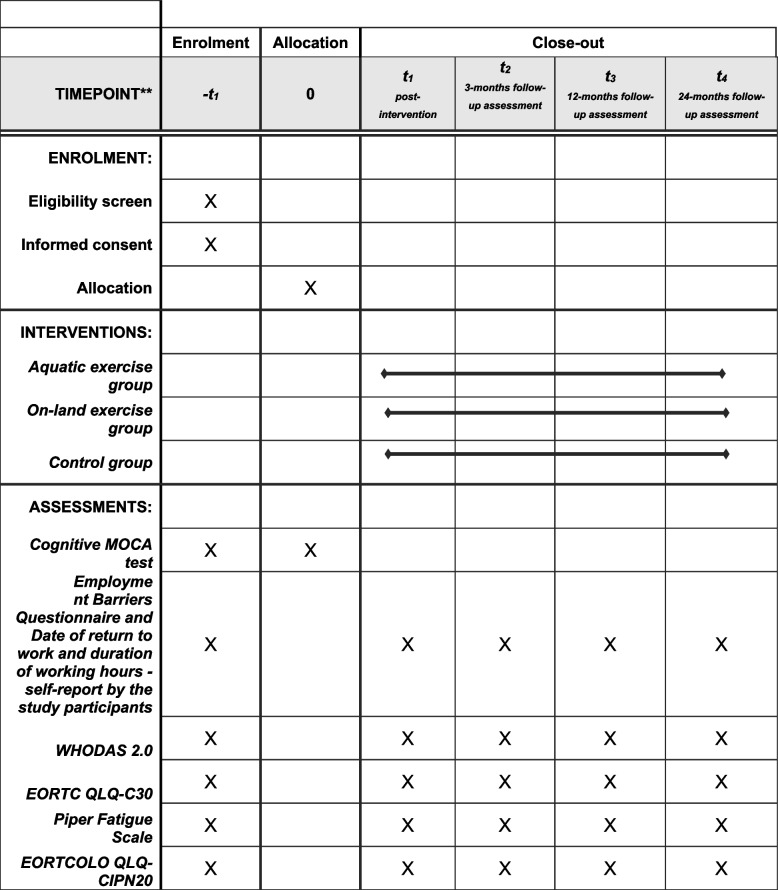
Content for the schedule of enrolment, interventions, and assessments

The schedule of enrolment, interventions, and assessments are shown in Fig. 2.

### Sample size {14}

One hundred and fifty cancer patients aged 18–65 years (stage III colon cancer patients and breast cancer patients with lymph node involvement) will be recruited via advertisements in Hadassah Medical Center and Tal Aviv University. The sample size is based on data from the WHO Disability Assessment Schedule (WHODAS 2.0), which has been used internationally to assess function and disability among a healthy population versus an unhealthy population [26]. The expected difference in the questionnaire scores between the two groups is 15 points and the average standard deviation is 15.08. The sample calculation includes a type 1 error of alpha equal to 0.05 with a power of 80%. According to this calculation, 16 participants are needed in each of the three study groups. Because we anticipate the withdrawal of some of the participants and because of the clinical condition of all participants, a number of 50 participants per group was selected.

### Recruitment {15}

Participants in this study will be recruited via flyers on social media (Facebook and Instagram), from references from colleagues (university faculty members, clinicians, and researchers), and via flyers/posters on Hadassah Medical Center.

### Assignment of interventions: allocation

#### Sequence generation {16a}

Simple randomization will be used in this study. Participants who met the inclusion criteria will be randomized to one of three groups, using specific online software (https://www.randomizer.org/). Ratio allocation will be 1:1:1 (approximately).

### Concealment mechanism {16b}

Intervention group assignment to participants or therapists cannot be concealed due to the nature of interventions. The allocation will be made by a member of the research team who will not be involved in the screening or group intervention.

### Implementation {16c}

A member of the research team who will not be involved in patient recruitment, assessment, or delivering the intervention will perform randomization and allocation.

### Assignment of interventions: Blinding

#### Who will be blinded {17a}

Due to the nature of this intervention, treatment allocation cannot be blinded to the participants or to the caregivers. Participants receive an explanation about the type of intervention, and caregivers are aware that everyone is a patient and know what type of treatment they provide.

### Procedure for unblinding if needed {17b}

This trial is not blinded to the participants or caregivers.

### Data collection and management

#### Plans for assessment and collection of outcomes {18a}

Most of the outcomes are collected in the hospital by questionnaires. In cases where participants do not come to a follow-up visit, data will be collected by phone interview.

#### Plans to promote participant retention and complete follow-up {18b}

Upon completion of the intervention, participants will be contacted by e-mail and phone calls to encourage them to complete the follow-up.

### Data management {19}

All data obtained will be at a secured platform. Patients will accept the informed consent and will be contacted by a researcher data collection meeting. The members of the research team in charge of the data collection will be trained. Data will be stored in a secure office at the Hadassah Medical Center. After that, patients’ data will be anonymized by a unique ID code on the dataset and will be stored in a secure server and the computer will have a password to be accessed only by the principal investigators from Tel Aviv University.

### Confidentiality {27}

Before entering the study, participants will complete the initial screening and the informed consent. Participants’ information will be encoded with a unique ID number to maintain confidentiality. The database will be protected on a computer with password security on a secured platform. Only the principal investigator from Tel Aviv University will have access to the data.

### Plans for collection, laboratory evaluation and storage of biological specimens for genetic or molecular analysis in this trial/future use {33}

No biological samples will be collected.

### Statistical methods

#### Statistical methods for primary and secondary outcomes {20a}

Every participant will be assigned a random study identifier number, ensuring the privacy of their personal data. The statistical analyses will be conducted in a way that prevents the identification of individual patients. We will conduct a series of mixed variance analyses to identify relationships and associations between groups, as well as changes over time within each group (independent variables). The dependent variables will be primary and secondary outcomes, and a separate analysis will be performed for each variable.

Additionally, we will conduct various analyses to explore the relationships between changes in the dependent variables.

### Interim analyses {21b}

No interim analysis will be carried out in this trial.

### Methods for additional analyses (e.g. subgroup analyses) {20b}

Additional analyses have not been planned.

### Methods in analysis to handle protocol non-adherence and any statistical methods to handle missing data {20c}

To address protocol non-adherence, intervention physiotherapists will receive intervention protocol. Missing data will be imputed using the random forest method.

### Plans to give access to the full protocol, participant level-data and statistical code {31c}

The principal investigator will provide access to the anonymized data upon reasonable request.

### Oversight and monitoring

#### Composition of the coordinating centre and trial steering committee {5d}

Principal investigator and members of the research team will oversee and responsible for monitoring the research.

### Composition of the data monitoring committee, its role and reporting structure {21a}

No harm to participants is expected.

### Adverse event reporting and harms {22}

In this study, no harm or adverse events are anticipated. However, in case of any adverse events, principal investigator will take appropriate actions to address them.

### Frequency and plans for auditing trial conduct {23}

The research team meets weekly to monitor the study and its procedures.

### Plans for communicating important protocol amendments to relevant parties (e.g. trial participants, ethical committees) {25}

All changes in the protocol must be approved by the Tel-Aviv University Ethics Committee and the Helsinki Committee of Hadassah Medical Organization, Israel. If any changes to the protocol are required during this trial, a new protocol will be designed and submitted for approval before implementation.

### Dissemination plans {31a}

Results of this study will be disseminated in: (1) scientific conferences, and (2) peer-reviewed indexed journals.

## Discussion

The popularity of exercise is increasing among cancer patients. Previous studies have reported the beneficial effects of exercise on exhaustion and fatigue [10], neuropathy [11], and overall quality of life [12] among cancer patients. However, few studies have specifically examined the impact of aquatic exercises [20, 21]. The existing studies suffer from low methodological quality, making it difficult to draw definitive conclusions regarding the true therapeutic effect of aquatic exercise.

This study aims to address this gap by conducting the first randomized controlled trial to investigate the effects of an exercise program conducted in water, compared to an identical program on-land, as well as a non-interventional exercise program, on cancer patients. The study will assess the levels of exhaustion, fatigue, and neuropathy, which are symptoms that significantly impact patients' daily activities, quality of life, and ability to return to work. By restoring the ability of cancer patients to fully engage in daily activities, it is expected that this study will promote the potential for their earlier return to work, ultimately reducing their reliance on social and economic support. The implications of this study on socio-economic policies are significant, as the results may offer a convenient, cost-effective, and non-invasive intervention program that can alleviate exhaustion, fatigue, and neuropathy, leading to improved quality of life and increased participation in daily activities, including a faster return to work. An expedited return to work and reduced dependence on social services would have both personal and public importance.

## Trial status

This is the first version of the protocol (registered 22 June 2022). The recruitment process started on April 01, 2023, and is expected to be completed in August 2027.

### Supplementary Information

**Additional file 1.**

## Data Availability

Only members of the research team will have access to the data. For the data from this study please contact MN.

## Abbreviations

WHODAS 2.0: The World Health Organization Disability Assessment Schedule
EORTC QLQ-C30: The European Organization for Research and Treatment of Cancer core quality of life questionnaire
EORTCOLO QLQ-CIPN20: The European Organization for Research and Treatment of Cancer Quality of Life Questionnaire–Chemotherapy-Induced Peripheral Neuropathy 20-item scale
