# Supplementary material for: Effects of aquatic exercise program versus on-land exercise program on cancer-related fatigue, neuropathy, activity and participation, quality of life, and return to work for cancer patients: study protocol for a randomized controlled trial
Source: BMC Complement Med Ther. 2024 Feb 2;24:74. doi: 10.1186/s12906-024-04367-8 (PMC10835900; doi:10.1186/s12906-024-04367-8)
Supplement: Supplementary file 1 — Additional file 1. [file 12906_2024_4367_MOESM1_ESM.docx]

**Administrative information**

*Trials* guidance: please include this text in your protocol just above the Administrative information table:

Note: the numbers in curly brackets in this protocol refer to SPIRIT checklist item numbers. The order of the items has been modified to group similar items (see <http://www.equator-network.org/reporting-guidelines/spirit-2013-statement-defining-standard-protocol-items-for-clinical-trials/>).

| Title {1} | Effects of aquatic exercise program versus on-land exercise program on cancer-related fatigue, neuropathy, activity and participation, quality of life, and return to work for cancer patients: study protocol for a randomized controlled trial |
| --- | --- |
| Trial registration {2a and 2b}. | ClinicalTrials.gov ID: NCT05427344, Registered 22 June 2022 |
| Protocol version {3} | V01 |
| Funding {4} | The study is funded by the National Insurance Institute of Israel |
| Author details {5a} | Michal Nissim, Teachers for Students with Complex and Multiple Disabilities track, The David Yellin Academic College of Education, Jerusalem, Israel.  Yakir Rottenberg, Department of Oncology, Hadassah Medical Organization and Faculty of Medicine, Hebrew University of Jerusalem, Israel.  Naama Karniel, Physiotherapy Department at Hadassah Medical Organization, Jerusalem, Israel.  Navah Z. Ratzon, Sackler Faculty of Medicine, School of Health Professions, Department of Occupational Therapy, Tel Aviv University, Israel. |
| Name and contact information for the trial sponsor {5b} | National Insurance Institute  217 Jaffa Street, Jerusalem 9199908 |
| Role of sponsor {5c} | The funding body will have no role in the design of the study or at any stage of it. |
